# HNF4α is a novel regulator of intestinal glucose-dependent insulinotropic polypeptide

**DOI:** 10.1038/s41598-019-41061-z

**Published:** 2019-03-12

**Authors:** Romain Girard, Mathieu Darsigny, Christine Jones, Faïza Maloum-Rami, Yves Gélinas, André C. Carpentier, Mathieu Laplante, Nathalie Perreault, François Boudreau

**Affiliations:** 1 Department of Anatomy and Cell Biology, Faculty of Medicine and Health Sciences, Université de Sherbrooke, Sherbrooke, QC Canada; 2 Centre de recherche de l’Institut universitaire de cardiologie et de pneumologie de Québec, Faculty of Medicine, Université Laval, Québec, QC Canada; 3 Department of Medicine, Division of Endocrinology, Faculty of Medicine and Health Sciences, Université de Sherbrooke, Sherbrooke, QC Canada

## Abstract

Mutations in the *HNF4A* gene cause MODY1 and are associated with an increased risk of Type 2 diabetes mellitus. On the other hand, incretins are hormones that potentiate reductions in blood glucose levels. Given the established role of incretin-based therapy to treat diabetes and metabolic disorders, we investigated a possible regulatory link between intestinal epithelial HNF4α and glucose-dependent insulinotropic polypeptide (GIP), an incretin that is specifically produced by gut enteroendocrine cells. Conditional deletion of HNF4α in the whole intestinal epithelium was achieved by crossing *Villin*-Cre and *Hnf4*α^*loxP/loxP*^ C57BL/6 mouse models. GIP expression was measured by qPCR, immunofluorescence and ELISA. Gene transcription was assessed by luciferase and electrophoretic mobility shift assays. Metabolic parameters were analyzed by indirect calorimetry and dual-energy X-ray absorptiometry. HNF4α specific deletion in the intestine led to a reduction in GIP. HNF4α was able to positively control *Gip* transcriptional activity in collaboration with GATA-4 transcription factor. Glucose homeostasis and glucose-stimulated insulin secretion remained unchanged in HNF4α deficient mice. Changes in GIP production in these mice did not impact nutrition or energy metabolism under normal physiology but led to a reduction of bone area and mineral content, a well described physiological consequence of GIP deficiency. Our findings point to a novel regulatory role between intestinal HNF4α and GIP with possible functional impact on bone density.

## Introduction

Hepatocyte nuclear factor - 4 alpha (HNF4α) is a transcription factor that belongs to the steroid/thyroid hormone receptor superfamily originally identified as a liver-enriched transcription factor but also expressed in gastrointestinal epithelia, the pancreas and kidneys^1–3^. HNF4α supports the morphogenetic development of the visceral ectoderm as evidenced by deletion of *Hnf4a* leading to early mouse embryonic death^4^. This observation has led to the design of conditional deletion strategies for *Hnf4a* in order to timely define its precise role in various organs during development. For instance, early embryonic hepatic loss of HNF4α has been shown to severely impair hepatocyte differentiation and function leading to premature death^4,5^ whereas deletion in adulthood resulted in viability with important hepatic dysfunctions^6^. Similarly, conditional deletion of *Hnf4a* in the colonic epithelium at a period of time preceding the gut embryonic cytodifferentiation program disrupted colon morphogenesis^7^ whereas deletion occurring after this developmental transition only partially affected intestinal epithelial homeostasis^8^. It is therefore assumed that HNF4α acts as a morphogen during early embryonic development rather than being systematically involved in epithelia maintenance.

The pathological consequence of *HNF4A* mutations was first associated with a hereditary form of diabetes referred to as mature-onset diabetes of the young 1 (MODY1)^9^. Among the 13 monogenic forms of diabetes, MODY1 particularly evolved from neonatal hypoglycemia to impaired glucose-stimulated insulin secretion (GSIS) and hypoinsulinemia-induced hyperglycemia without being associated with β-cell autoimmunity or obesity phenotypes in humans^10^. In mouse models, the targeted loss of *Hnf4α* in pancreatic β-cells was not sufficient to cause hypoinsulinemia and there are conflicting reports as to whether HNF4α is crucial in sustaining GSIS in these murine models^11,12^. These observations suggest that other HNF4α-defective tissues could be involved to fully recapitulate MODY1 pathogenesis^11,12^.

HNF4α controls metabolism in the liver where it regulates glycolytic enzymes and glucose transporters^13^ as well as lipid homeostasis^6^. As a nuclear receptor, HNF4α harbors ligand-binding affinities for medium to long chain fatty acids and their metabolites that are capable of modulating its transcriptional activity^14–16^. Given that MODY1 patients display lower circulating triglycerides as part of their pathological phenotype, the hepatic contribution of HNF4α to this metabolic disorder is suspected, although remains to be clarified^17,18^. The intestinal epithelium represents another crucial tissue regulating whole organism metabolism. To this end, the enterocytes forming the intestinal barrier act as one of the first regulated steps of nutrient absorption and subsequent delivery. Previous observations support a functional role for HNF4α in interfering with these processes. Cultured intestinal epithelial cells made deficient in HNF4α were found less efficient in cellular lipid transport^19^. Additionally, conditional deletion of *Hnf4a* in the murine intestine impacted enterocytic fatty acid uptake after lipid gavage^20^. On the other hand, enteroendocrine cells from the intestinal epithelium produce a wide range of peptides mediating digestive rate, bone remodeling, appetite, GSIS, adipogenesis and global energy homeostasis control. Among these various peptides, incretins that comprise intestinal specific glucose-dependent insulinotropic polypeptide (GIP) actively contribute to GSIS by enhancing insulin secretion upon glucose sensing by pancreatic β-cells, and are also involved in a number of other biological activities including bone metabolism^21^. The present study aimed to investigate whether intestinal epithelial HNF4α influences GIP regulation, glucose homeostasis and metabolism in mice. We report herein that conditional deletion of *Hnf4a* in the intestinal murine epithelium significantly reduces GIP production, impacts bone density but does not influence whole body energy metabolism under normal physiological conditions.

## Materials and Methods

### Animals and analytical procedures

C57BL/6J-*Hnf4*α^*loxP/*+^ mice^8^ were first crossed with C57BL/6-*12.4KbVil*Cre mice^22^ to generate *12.4KbVil*Cre/*Hnf4*α^*loxP/*+^ mice which were subsequently bred with *Hnf4*α^*loxP/loxP*^ mice to produce *12.4KbVil*Cre/*Hnf4*α^*loxP/loxP*^ (HNF4α^ΔIEC^) mutant mice and their controls. Mice were genotyped as previously described^8,23,24^ and treated in accordance with a protocol reviewed and approved by the Institutional Animal Research Review Committee of the Université de Sherbrooke (approval ID number 102–18) and in accordance with relevant guidelines and regulations. All experiments were carried out using male mice maintained on chow diet. Blood glucose values were determined from whole venous blood from fasted mice or during glucose tolerance tests as previously described^25^. Glucose tolerance tests were performed *per os* (OGTT) or intraperitoneally (IPGTT) both with a 2 mg/g by weight glucose dose, while insulin tolerance test (ITT) was assessed with a 0.75 mIU/g by weight dose. The following mouse serum hormone levels were measured using rat/mouse ELISA kits according to the manufacturer’s instructions: Total GIP (EMD Millipore, EZRMGIP-55K); GLP-1 (Crystal Chem, 81508) and Insulin (Crystal Chem, 90080). For metabolic analyses, mice were placed in metabolic cages as described previously^25^. All groups were fed ad libitum throughout the duration of the study. Following a 5-day adaptation period, body weight (g), food intake (g), water intake (ml) as well urine (ml) and feces (g) output were measured daily at the same hour. Fecal pellets were collected freshly and residual gross energy density was determined on dried samples using adiabatic bomb calorimetry (Parr Instruments, Moline, IL, USA). Body composition on post-mortem mice was measured by dual-energy X-ray absorptiometry (DEXA) using the PIXIMUS mouse densitometry apparatus (Lunar Corporation, Madison, WI, USA).

### Indirect calorimetry

The Promethion High-Definition Room Calorimetry System was used for indirect calorimetry studies (GA3, Sable Systems. Las Vegas, NV). Data acquisition and instrument control were coordinated by MetaScreen v. 1.6.2 and the obtained raw data was processed using ExpeData v. 1.4.3 (Sable Systems, Las Vegas, NV) using an analysis script detailing all aspects of data transformation. A standard 12 h light/dark cycle (6:00–18:00) was maintained throughout the calorimetry studies. Prior to data collection, all animals were acclimated to cages for 3 days followed by 4 days of data acquisition^26^. The derived Weir’s equations^27^ revised by the non-protein assumption^28^ were used to estimate mouse oxidative rates for carbohydrates (4.585 CO_2_–3.226 VO_2_ (mg/min/kg body weight)) and fat (1.695 VO_2_–1.701 CO_2_ (mg/min/kg body weight)).

### RNA isolation and qRT-PCR

Total RNA from the jejunum and colon was isolated and qRT-PCR was performed as previously described^29,30^. Target expression was quantitated relatively to TATA box binding protein (Tbp) gene expression. Primer sequences used for qPCR were as follows: Hnf4a sense: 5′-GTGCTGCTCCTAGGCAATGA-3′; Hnf4a antisense: 5′-ACTCAGCCCCTTGGCATCT-3′; Gip sense: 5′-GGCTAGGGGACACAATCTAGG-3′; Gip antisense: 5′-GGATCGGAACTCAACCTCTTC-3′; Gcg sense: 5′-TGATGAACACCAAGAGGAACC-3′; Gcg antisense: 5′CCTGGCCCTCCAAGTAAGA-3′; Tbp sense: 5′GGGGAGCTGTGATGTGAAGT-3′; Tbp antisense: 5′-GGAGAACAATTCTGGGTTTGA-3′.

### Immunofluorescence

Mouse pancreas, duodenum, jejunum, ileum and colon biopsies were fixed in 4% paraformaldehyde overnight at 4°C, dehydrated, embedded in paraffin and cut in 5-µm sections. Immunofluorescence studies were performed as previously described^25^. The following primary affinity-purified antibodies were used: goat anti-GIP (Santa Cruz, sc-23554; diluted 1/200), mouse anti-GLP-1 (Santa Cruz, sc-57166, diluted 1/200), mouse anti-insulin (Santa Cruz, sc-8033, diluted 1/50) and mouse anti-glucagon (Santa Cruz, sc-514592, diluted 1/300). Alexa 488 (Cell Signaling Technology; #4408; diluted 1/400) and Alexa 594 (Cell Signaling Technology; #8890; diluted 1/400) were used as secondary antibodies. Stained sections were analyzed using the NanoZoomer 2.0-RS (Hamamatsu Photonics, Japan) digital slide scanner, the LX2000 fluorescence module (Hamamatsu Photonics, Japan) and NDP.View software.

### Plasmid construction, cell culture and luciferase assays

The −192 to +39 region of the mouse *Gip* promoter was amplified by PCR from purified genomic DNA isolated from C57BL/6 mouse tail with the following primers: 5′-GCCCCAGATAACGCTAGAGA-3′ and 5′-TCTTCTCCTCCTACCTGTTGG-3′. The amplicon was subcloned into the pGL3basic luciferase reporter vector (Promega, Madison, WI). Mutagenesis of the *Gip* promoter constructs for the HNF4α site (H1) and the GATA site 1 (G1) was performed with the QuickChange Lightning site-directed mutagenesis kit (Agilent Technologies, Santa Clara, CA) while mutagenesis of the GATA site 2 (G2) was performed by GenScript custom services (Piscataway, NJ). The respective integrities of subcloned PCR and mutagenesis products were all confirmed by sequence analysis. 293 T cells were plated in 24-well plates and transfected with 200 ng of wild-type or mutated *Gip* promoter luciferase constructs, 5 ng of the phRL-CMV Renilla luciferase vector (Promega, Madison, WI), a combination of 100 ng of pBabepuro/HNF4α1^8^ and 100 ng of pBabepuro/GATA-4^31^ expression vectors with a constant total DNA amount of 800 ng per transfected well, and 1.6 µl of Lipofectamine 2000 for a total of 100 µl of OptiMEM (Life Technologies Inc, Burlington, ON). The medium was replaced after 4 h by fresh DMEM supplemented with 10% FBS. Luciferase and Renilla activities were determined 48 h after the transfection with the dual luciferase assay kit (Promega, Madison, WI). Each experiment was repeated three times in triplicate.

### EMSA

Electrophoretic mobility shift assays (EMSA) were performed as described previously^32^. The reactions were performed with 5 µg of nuclear protein extracts from 293 T cells transfected or not with pBabepuro/GATA4 or pBabepuro/HNF4α1 expression vectors. For the supershift analysis, 200 ng of HNF4α C-19 (sc-6556 X) antibody, 200 ng of GATA4 C-20 (sc-1237 X) or 200 ng of irrelevant HNF1α C-19 (sc-6547 X) (Santa Cruz Biotechnology, Santa Cruz, CA, USA) were added and the binding reactions were continued for 10 minutes at room temperature. Retarded complexes were then separated on a 5% polyacrylamide gel at 4 °C during 4 hours, dried for 1 hour at 80 °C and exposed on autography film. The DNA probes consisted of double-stranded oligonucleotides of individual binding sites for both HNF4α (H1) and GATA4 (G1 and G2) within the promoter region of the *Gip* gene (MatInspector software tool, http://www.genomatix.de)^33^. Positive controls for GATA4 and HNF4α binding were used from the *Sis* gene promoter^34^ and *APOC3* gene promoter^23^.

### Statistical analysis

**Figure 1:**
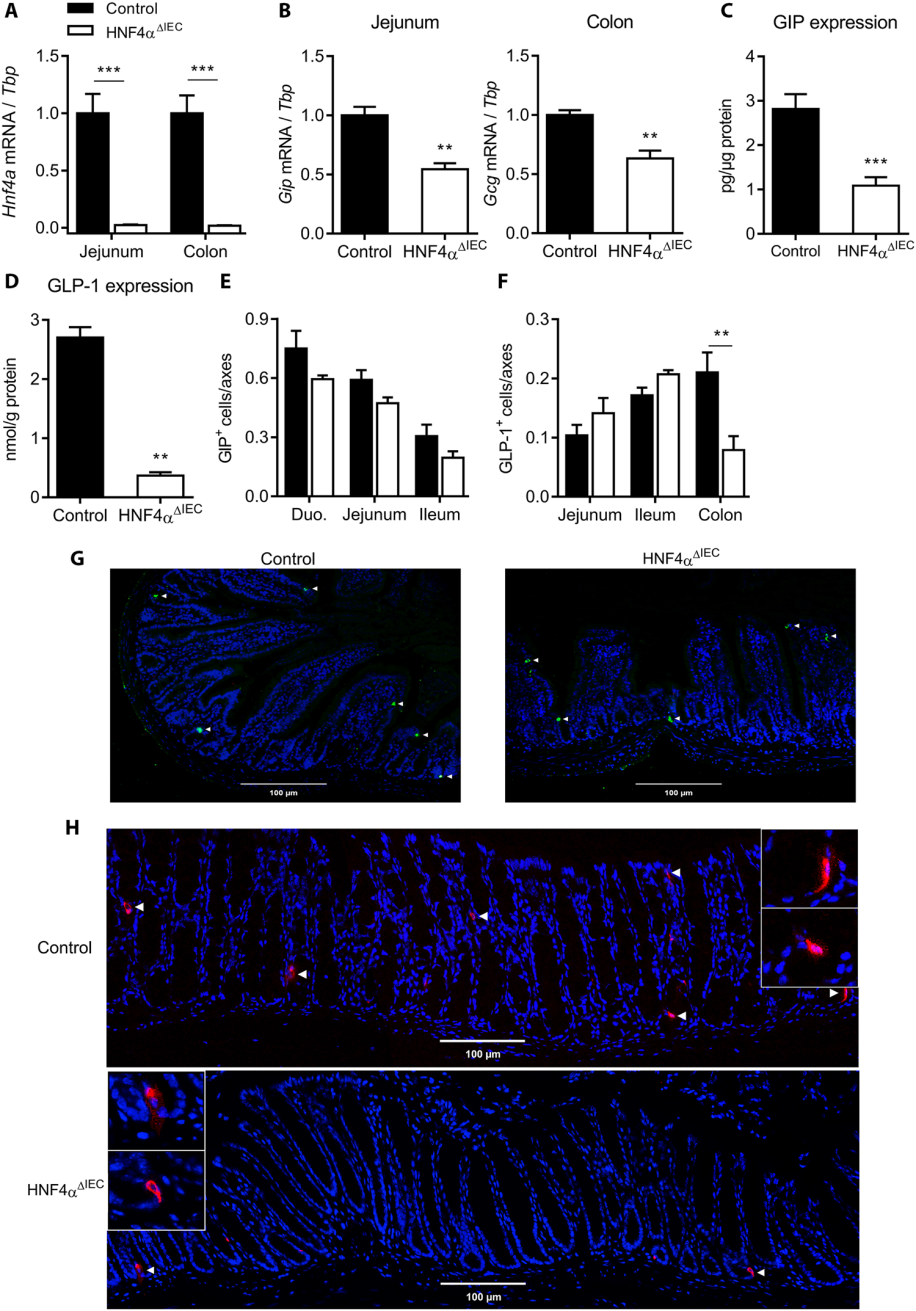
HNF4α regulates GIP and GLP-1 expression in the mouse intestine. (**A**) *Hnf4a* gene expression relative to *Tbp* measured in the jejunum and colon of 1 week-old control (black columns) and HNF4α^ΔIEC^ (white columns) mice (n = 4–6). (**B**) *Gip* and *Gcg* gene expression relative to *Tbp* measured in the jejunum and colon of control (black columns) and HNF4α^ΔIEC^ (white columns) mice (n = 4–8). (**C**) GIP protein level quantified by ELISA in the jejunum of control (black columns) and HNF4α^ΔIEC^ (white columns) mice (n = 5–10). (**D**) GLP-1 protein level quantified by ELISA in the colon of control (black columns) and HNF4α^ΔIEC^ (white columns) mice (n = 5–10). Enteroendocrine cells immunopositive for GIP (**E**) or GLP-1 (**F**) were counted per crypt-villus axes along the intestinal tract from both control (black columns) and HNF4α^ΔIEC^ (white columns) mice (n = 3). Representative immunostaining of enteroendocrine cells expressing GIP in the duodenum (**G**) and GLP-1 in the colon (**H**) of control and HNF4α^ΔIEC^ mice.

**Figure 2:**
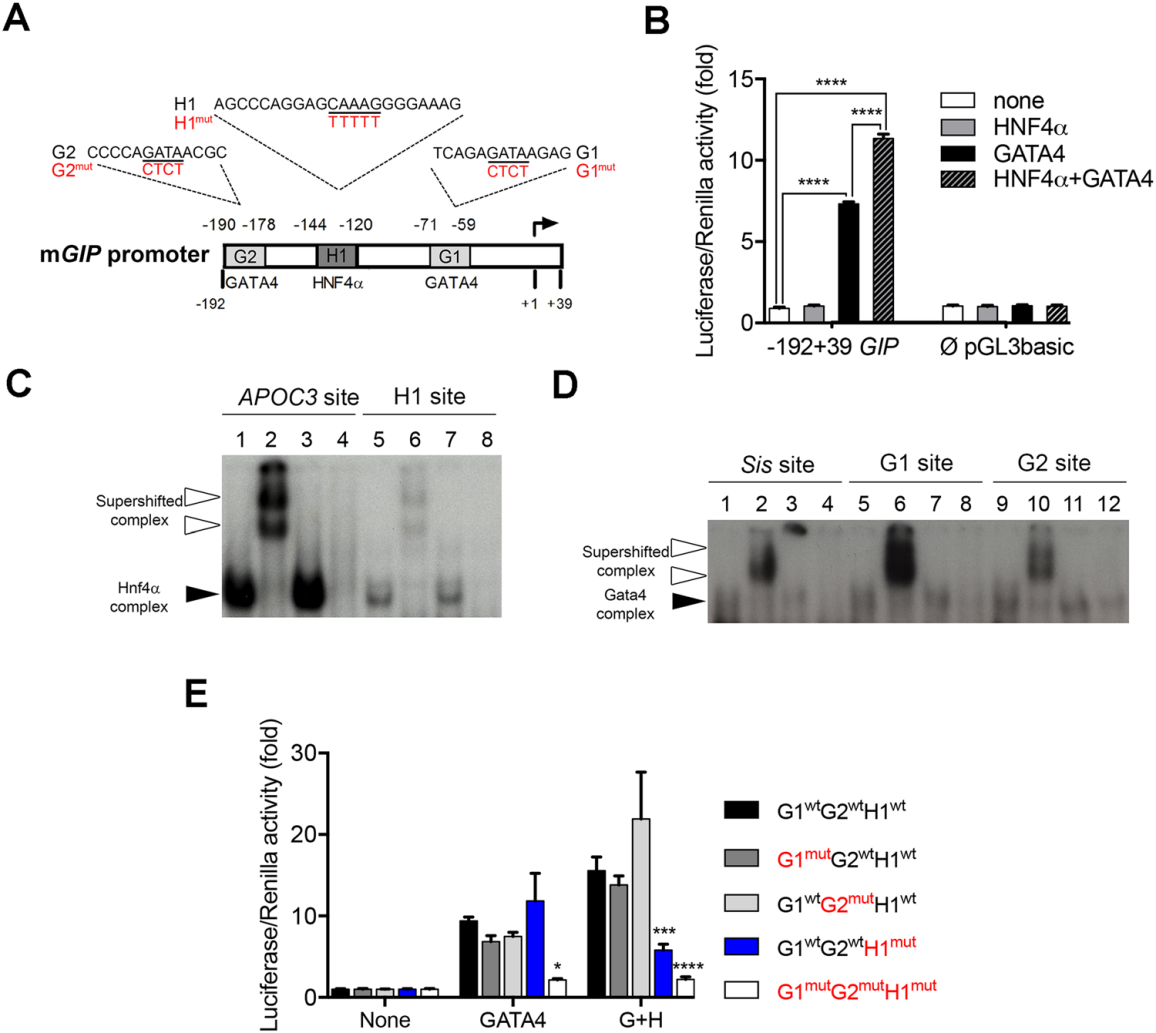
The mouse *Gip* promoter is regulated by HNF4α. (**A**) Schematic representation of the mouse *Gip* promoter with predicted binding sites for HNF4α and GATA4. Mutations introduced in these sites are indicated in red. (**B**) Luciferase assay using the −192 +39 *Gip*-PGL3basic and Empty-pGL3basic constructs with a combination of HNF4α and/or GATA4 expression vectors. Luciferase assays are representative of three independent.experiments and are presented as mean ± SEM; ****P < 0.0001. (**C**) EMSA analysis with HNF4α or (**D**) GATA4 nuclear extracts of each potential binding site. Lines 1, 5 and 9 show ^32^P-labelled probes with nuclear extracts; lines 2, 6 and 10 show ^32^P-labelled probes with nuclear extracts and antibodies; lines 3, 7 and 11 show ^32^P-labelled probes with nuclear extracts and irrelevant antibodies; lines 4, 8 and 12 show ^32^P-labelled probe with nuclear extracts from non-transfected empty cells. White arrowheads point to supershifted complexes while black arrowheads depict retarded complexes. Full length gels of these analyses are provided in Supplementary Fig. 2. (**E**) Luciferase assay using the −192 +39 *Gip*-PGL3 wild-type or mutated promoter constructs in combination of HNF4α (H) and/or GATA4 (G) expression vectors. Mutation effects on luciferase activity are evaluated against the wild-type promoter for each condition. Luciferase assays are representative of three independent experiments and are presented as mean ± SEM; *P < 0.05, ***P < 0.001, ****P < 0.0001.

**Figure 3:**
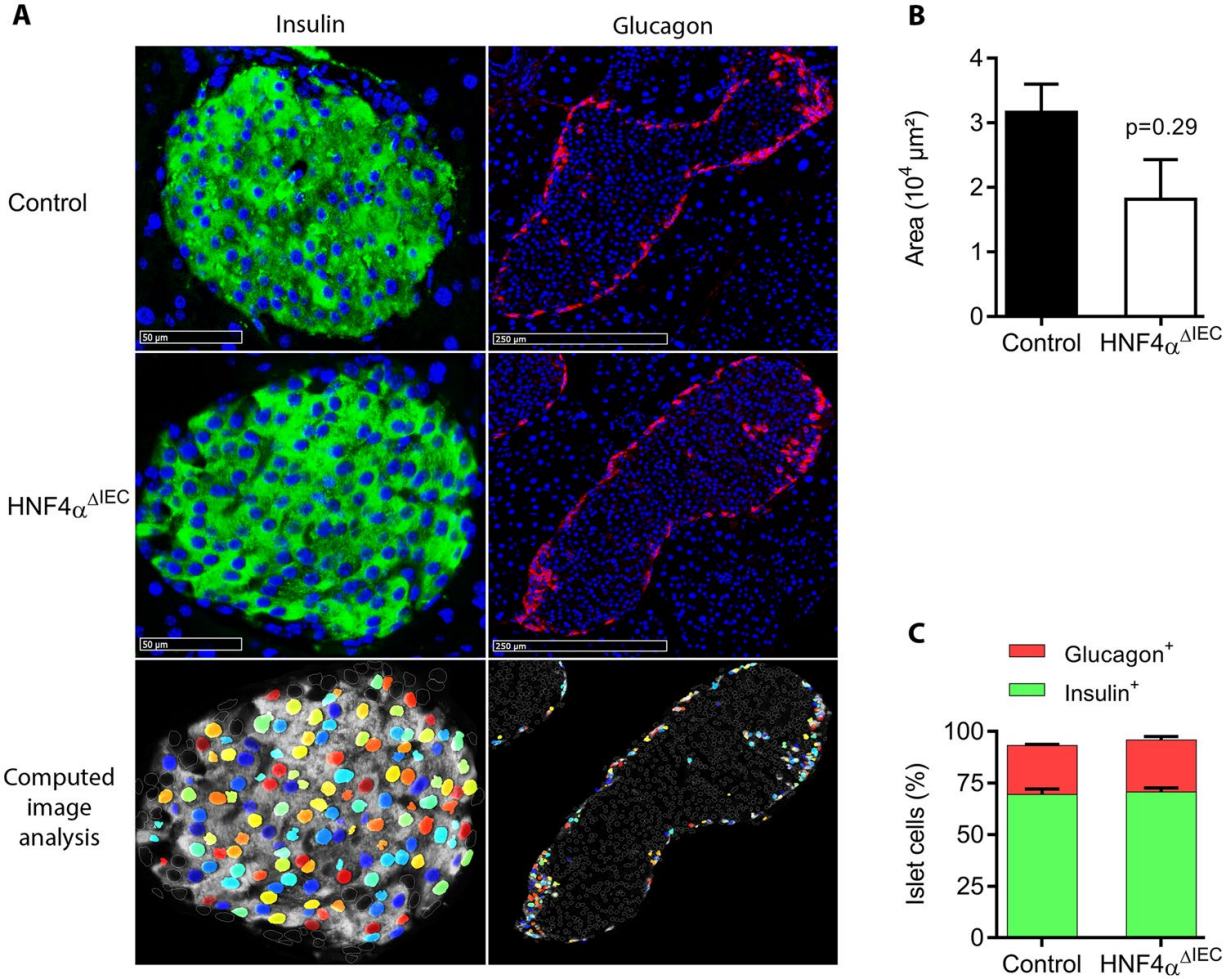
Pancreatic islet structures of HNF4α^ΔIEC^ mice. (**A**) Representative immunofluorescence of insulin and glucagon on two distinct islets of Langerhans sections obtained from adult control and HNF4α^ΔIEC^ mice. Computed image analysis using CellProfiler (3.1.5) was designed to quantify labeled cells. Representative image analysis obtained from HNF4α^ΔIEC^ mice islet sections (bottom panel) highlights positive detected cells (colored nuclei), overlapping immunofluorescence (white diffuse staining) and non-labeled islet cells (empty and white circled nuclei). (**B**) Pancreatic islets area measured in adult control (black column) and HNF4α^ΔIEC^ (white column) mice (n = 4–5). (**C**) Relative population of cells positive for insulin (green columns) or glucagon (red columns) signals in pancreatic islets of adult control and HNF4α^ΔIEC^ mice (n = 3–5).

**Figure 4:**
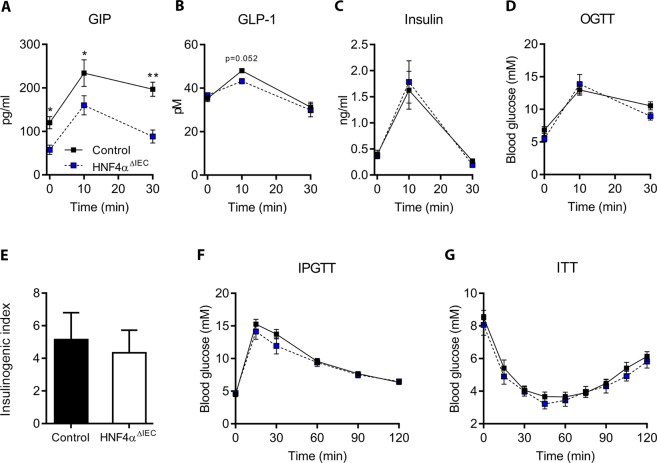
Hormonal and glycemic response in HNF4α^ΔIEC^ mice. Circulating GIP (**A**), GLP-1 (**B)** and insulin (**C**) levels measured by ELISA in control (black squares) and HNF4α^ΔIEC^ (blue squares) mice following an oral glucose tolerance test (OGTT) (n = 4–10). (**D**) Blood glucose measured in control (black squares) and HNF4α^ΔIEC^ (blue squares) mice during OGTT (n = 4 = 10). (**E**) Insulinogenic index was calculated in control (black columns) and HNF4α^ΔIEC^ (white columns) mice as follows: Δinsulin peak/Δblood glucose peak, where Δ is the time point concentration minus baseline level (n = 6–7). (**F**) Blood glucose measured in control (black squares) and HNF4α^ΔIEC^ (blue squares) mice during an intraperitoneal glucose tolerance test (IPGTT) (n = 7–9). (**G**) Insulin tolerance test (ITT) performed on control (black squares) and HNF4α^ΔIEC^ (blue squares) mice (n = 6–11).

**Figure 5:**
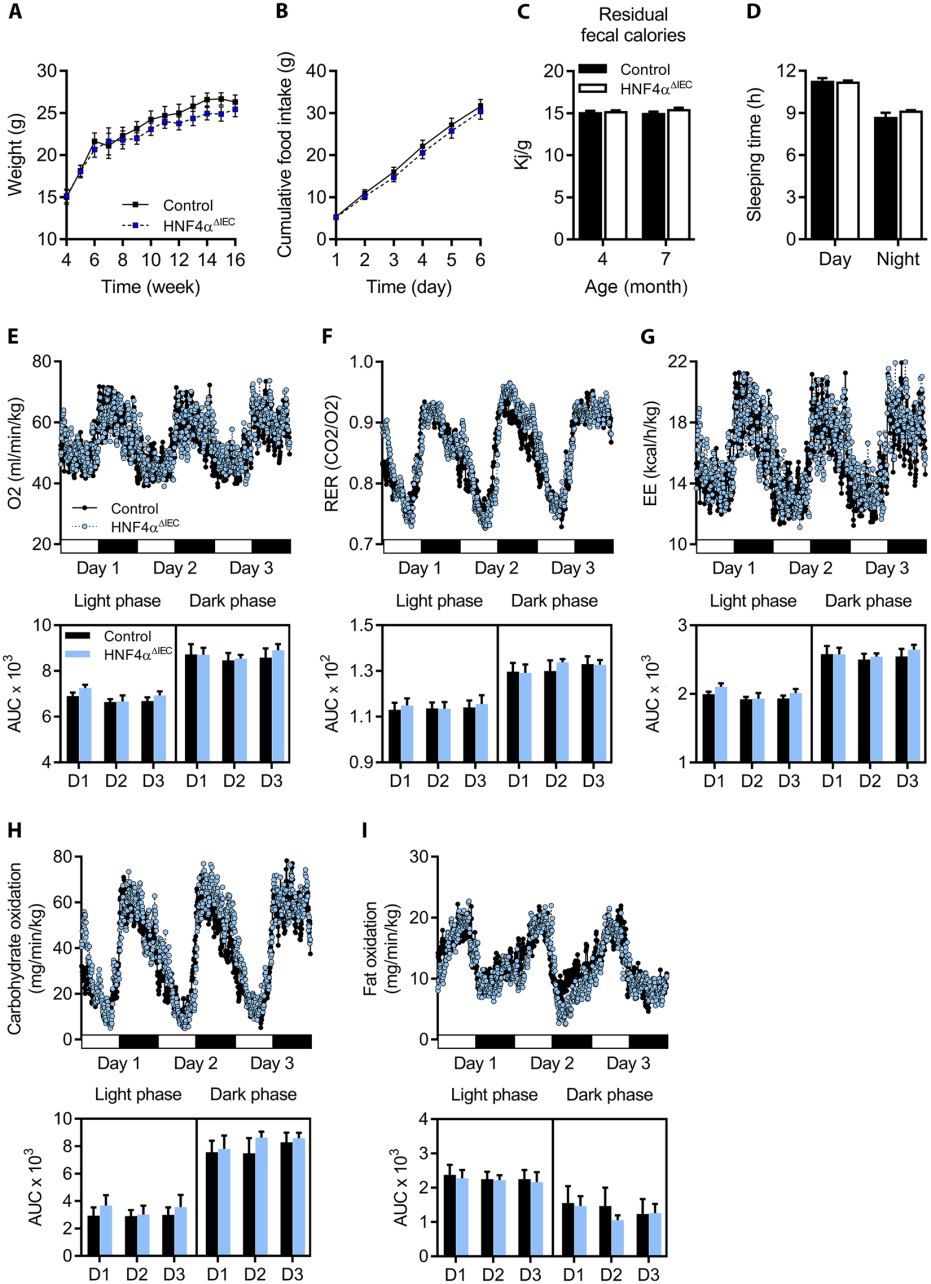
Nutrition behaviors and energy metabolism in HNF4α^ΔIEC^ mice. (**A**) Weight curves for adult control (black squares) and HNF4α^ΔIEC^ (blue squares) mice (n = 4–13). (**B**) Cumulative food intake was monitored in metabolic cages during 6 consecutive days for both adult control (black squares) and HNF4α^ΔIEC^ (blue squares) mice (n = 5–6). (**C**) Calorie density determined from fecal samples collected from 4-month-old and 7-month-old control (black columns) and HNF4α^ΔIEC^ (white columns) mice (n = 3–5). (**D**) Sleeping time monitored in metabolic cages and calculated as the mean of 3 consecutive days for both adult control (black columns) and HNF4α^ΔIEC^ (white columns) mice (n = 7). Oxygen consumption (**E**), respiratory exchange ratio (RER) (**F**), and energy expenditure (EE) (**G**) measurements acquired for 3 consecutive days for both adult control (black circles) and HNF4α^ΔIEC^ (blue circles) mice (n = 7) with corresponding area under the curve (AUC). Estimated oxidative rates for carbohydrates (**H**) and fat (**I**) with AUC calculated for 3 consecutive days for both adult control (black circles) and HNF4α^ΔIEC^ (blue circles) mice (n = 7).

**Figure 6:**
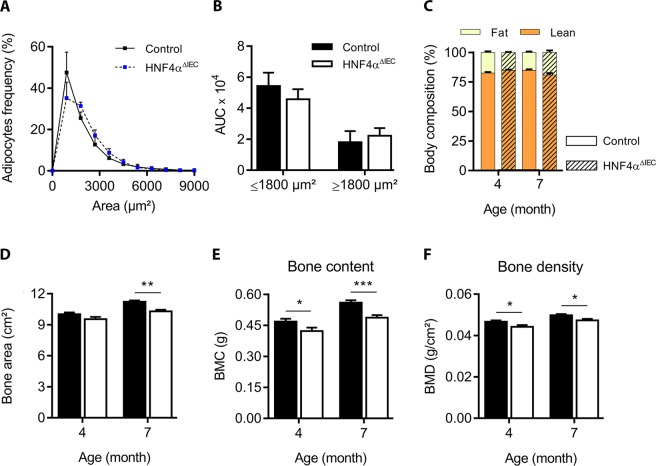
White adipocyte tissue density and body composition in HNF4α^ΔIEC^ mice. (**A**) Ranging of adipocytes according to their size as quantified by CellProfiler (3.1.5) from white adipose tissue sections from adult control (black squares) and HNF4α^ΔIEC^ (blue squares) mice (n = 7–8). The AUC calculated from control (black columns) and HNF4α^ΔIEC^ (white columns) mice (n = 7–8) is represented in (**B**). Dual-energy X-ray absorptiometry (DXA) measurements for body composition were acquired post-mortem from 4-month-old and 7-month-old mice. (**C**) Fat tissues (light yellow) and lean tissues (dark orange) are shown relative to total body weight of control (dark bordered squares) and HNF4α^ΔIEC^ (hatched squares) mice (n = 8–13). Osteodensitometry measurements for bone area (**D**), bone mineral content (BMC) (**E**) and bone mineral density (BMD) (**F**) of control (black columns) and HNF4α^ΔIEC^ (white columns) mice were calculated from DXA analysis (n = 8–13).

Statistical analyses were performed using GraphPad Prism 7 software. Figures 1B–D, 3B and 4E were analyzed using the Mann-Whitney test while 2-way ANOVA tests were used to analyze Figs 1A,E,F, 2B,E, 3C, 4A–D,F,G, 5 and 6. 2-way ANOVA tests were corrected for multiple comparisons using the Holm-Sidak method. Differences were considered significant with a *P* value of < 0.05. The data are presented as mean ± standard error of the mean; *P < 0.05, **P < 0.01, ***P < 0.001, ****P < 0.0001.

## Results

### Loss of intestinal HNF4α negatively impacts incretins production from the gut

Mice were conditionally deleted for *Hnf4*α in the intestinal epithelium as previously described^8,23,24^. Total RNA isolated from the jejunum and colon of 1 week-old HNF4α^ΔIEC^ and control mice were used to confirm that *Hnf4*α gene transcript expression was decreased in the intestinal epithelium of HNF4α^ΔIEC^ mice as determined by qRT-PCR (Fig. 1A). In order to evaluate a possible regulatory link between HNF4α and gut specific incretins, GIP and GLP-1 gene transcript expression was measured from the jejunum and the colon, respectively. A significant reduction was observed for jenunal *Gip* gene transcript (46%, *P* < 0.01) and colonic *Gcg* gene transcript (37%, *P* < 0.01) of 1 week-old HNF4α^ΔIEC^ mice (Fig. 1B). This reduction in *Gip* and *Gcg* transcripts was also maintained in adult HNF4α^ΔIEC^ mice (data not shown). To assess whether modulation at the gene transcript level impacted mature incretins production, ELISA essays were performed on intestinal tissues. GIP peptide concentration was reduced by more than 61% (*P* < 0.001) in the jejunum of fasted HNF4α^ΔIEC^ mice (Fig. 1C) while GLP-1 peptide concentration dropped by 87% (*P* < 0.01) in the colon of these mice (Fig. 1D). Immunofluorescence experiments were next performed to assess whether changes observed in incretin production was associated with variations in K (GIP) and L (GLP-1) enteroendocrine cell populations. As expected, the relative number of GIP^+^ cells was most abundant in the proximal intestine and gradually decreased toward the distal part while GLP-1^+^cells were more abundant in the distal intestine as previously reported elsewhere^35^. HNF4α^ΔIEC^ mice displayed non-significant changes in the relative number of GIP^+^ cells along the intestinal segments (Fig. 1E). Intriguingly, loss of intestinal HNF4α led to a reduction in the number of GLP-1^+^ cells in the colon (62%, *P* < 0.01) without affecting their number in both the jejunum and the ileum (Fig. 1F). Representative immunostaining results are shown for GIP^+^ cells within the duodenum (Fig. 1G) and GLP-1^+^ cells within the colon (Fig. 1H). Collectively, these observations suggest that HNF4α positively impacts *Gip* gene transcript and peptide expression, while mediating GLP-1 expression in a complex regional manner along the anterior-posterior axis of the gut epithelium.

### HNF4α regulates *Gip* promoter activity

Since the loss of HNF4α was found to correlate well with *Gip* gene transcript expression without majorly affecting GIP + cell production throughout the small intestine, *Gip* promoter was further screened for potential HNF4α DNA binding sites. A 193 bp region upstream of the transcription start site (TSS) of the murine *Gip* gene was analyzed since it was previously reported to represent the minimal promoter region required to direct specific expression in endocrine cells^36^. This analysis predicted one HNF4α binding site (H1) flanked by 2 GATA DNA binding sites (G1 and G2) (Fig. 2A). Interestingly, comparison of the *GIP* promoter among different species including human displayed a relatively well conserved distribution pattern of these GATA/HNF4α predicted binding sites (Supplementary Fig. 1). Co-transfection experiments with increasing concentrations of HNF4α did not impact −192 to +39 *Gip* promoter activity (data not shown). Since GATA-4 has been shown to regulate GIP expression^37^ and to physically interact with HNF4α^38^, cooperative interaction of both HNF4α and GATA-4 was tested on *Gip* promoter activity. GATA-4 alone was able to induce a greater than 7.3-fold increase (*P* < 0.0001) in *Gip* promoter activity while HNF4α alone had no effect (Fig. 2B). However, the combination of GATA4 and HNF4α led to a 11.3-fold (*P* < 0.0001) synergistic induction of the −192 to +39 *Gip* promoter construct (Fig. 2B). Both GATA-4 and HNF4α expression vector did not influence pGL3basic empty reporter activity (Fig. 2B). To further determine whether the predicted HNF4α and GATA-4 DNA binding sites of the *Gip* promoter were functional, EMSA was performed with double stranded ^32^P-labelled probes corresponding to these specific sites. Nuclear extracts isolated from 293 T cells transfected with an HNF4α expression vector were able to generate a complex with both the *Apoc3* HNF4α binding site used as a positive control (lane 1, Fig. 2C) and the *Gip* H1 site (lane 5, Fig. 2C) as opposed to empty nuclear extracts isolated from non-transfected 293 T cells (lanes 4 and 8, Fig. 2C). This complex was specific for HNF4α binding since inclusion of HNF4α antibodies led to the formation of supershifted complexes (lanes 2 and 6, Fig. 2C) which did not form upon substitution with irrelevant antibodies (lanes 3 and 7, Fig. 2C). A similar approach was also used with nuclear extracts isolated from 293 T cells transfected or not with a GATA-4 expression vector. A GATA-4 complex was observed for the *Sis* GATA binding site used as a positive control (lane 1, Fig. 2D), as well as for the *Gip* G1 (lane 5, Fig. 2D) and *Gip* G2 (lane 9, Fig. 2D) sites when GATA-4 positive nuclear extracts were used as opposed to empty 293 T nuclear extracts (lanes 4, 8 and 12, Fig. 2D). Again, this complex was specific for GATA-4 since the use of GATA-4 antibodies produced supershifted complexes (lanes 2, 6 and 10, Fig. 2D) as opposed to the use of irrelevant antibodies (lanes 3, 7 and 11, Fig. 2D). Mutagenesis of H1 (HNF4α), G1 (GATA) and G2 (GATA) sites was next achieved to monitor the importance of these sites for synergistic activation of the *Gip* promoter from both HNF4α and GATA-4. While mutagenesis of either G2 alone or G1 alone did not significantly impact the combined GATA-4 and HNF4α induction of *Gip* transcription, mutagenesis of H1 in the presence of both GATA-4 and HNF4α led to a 63% reduction in synergistic activation of the *Gip* promoter (*P* < 0.001) (Fig. 2E). Mutagenesis of all G1, G2 and H1 sites reduced the GATA-4 and HNF4α synergistic effect on *Gip* promoter transcription by more than 86% (*P* < 0.0001) (Fig. 2E). Collectively, these observations support that the interaction of HNF4α with its binding site is of functional importance to positively regulate *Gip* promoter activity.

### Loss of intestinal HNF4α does not influence glucose homeostasis or glucose-stimulated insulin secretion after oral or intraperitoneal glucose challenges

Given that incretins support pancreatic functions, we next monitored whether loss of intestinal HNF4α and associated reduction of intestinal GIP and GLP-1 production influenced pancreatic islets features. Both control and HNF4α^ΔIEC^ pancreatic islets displayed typical structures with inner insulin-expressing cells (β-cells) surrounded by glucagon-expressing cells (α-cells) (Fig. 3A). When compared to control mice, HNF4α^ΔIEC^ mice did not show significant change in islets size (Fig. 3B) as well as in β-cell and α-cell distribution (Fig. 3C). We then investigated whether the observed changes in gut incretins expression could impact circulating levels of both GIP and GLP-1 as well as glycemic parameters in HNF4α^ΔIEC^ mice following an oral glucose tolerance test (OGTT). Fasting circulating levels of GIP in HNF4α^ΔIEC^ mice were reduced by 52% when compared to control mice (*P* < 0.05) (Fig. 4A). OGTT increased GIP levels reaching a peak at 10 min and then decreasing at 30 min in both control and HNF4α^ΔIEC^ mice (Fig. 4A). However, the magnitude of the GIP response remained weaker in HNF4α^ΔIEC^ mice with a 32% reduction (*P* < 0.05) at 10 min and 55% reduction (*P* < 0.01) at 30 min in circulating GIP levels compared to controls. In contrast to GIP, fasting circulating levels of GLP-1 were not affected in HNF4α^ΔIEC^ mice (Fig. 4B). As expected, OGTT transiently increased GLP-1 levels at 10 min in both genotypes with a modest but not significant decrease of peak values for HNF4α^ΔIEC^ mice (Fig. 4B). Insulin release (Fig. 4C) and blood glucose concentration (Fig. 4D) remained unchanged between HNF4α^ΔIEC^ and control mice during OGTT, consistent with a similar insulinogenic index in these mice (Fig. 4E). In order to gain further insights into glycemic control in these mice, glucose and insulin tolerance tests were achieved intraperitoneally. Both glucose tolerance (Fig. 4F) and insulin tolerance (Fig. 4G) remained similar between HNF4α^ΔIEC^ and control mice. These data indicate a defective glucose-stimulated GIP response in HNF4α^ΔIEC^ mice that was not counteracted by GLP-1 and without influencing GSIS.

### Loss of intestinal HNF4α and coincidental GIP down-regulation do not impact nutrition and energy metabolism but negatively influence bone density

To gain further insight into the physiological impact of reduced GIP production in HNF4α^ΔIEC^ mice, metabolic parameters were next assessed. HNF4α^ΔIEC^ mice showed similar weight growth curves when compared to control mice (Fig. 5A), consistent with similar food intakes from both groups (Fig. 5B). Gross intestinal absorption ability remained similar in 4-month-old and 7-month-old HNF4α^ΔIEC^ and control mice as determined by total residual fecal caloric concentrations (Fig. 5C). Sleep and daily rhythms were also similar between HNF4α^ΔIEC^ and control mice (Fig. 5D) with no significant change in energy homeostasis as determined by oxygen consumption (Fig. 5E), respiratory exchange ratio (RER) (Fig. 5F) and energy expenditure (EE) (Fig. 5G). Oxidative rates fluctuations during light and dark phases for both carbohydrate (Fig. 5H) and fat (Fig. 5I) remained also similar between control and HNF4α^ΔIEC^ mice. Because incretins can regulate adipogenesis, we also assessed whether adipose density was affected in HNF4α^ΔIEC^ mice. Analysis of the white adipocyte tissue showed a similar frequency distribution of adipocyte sizes when compared between HNF4α^ΔIEC^ and control mice (Fig. 6A,B). DXA body composition analysis of young adults (4-month-old) and aging (7-month-old) HNF4α^ΔIEC^ and control mice also showed similar lean and fat mass among the groups (Fig. 6C). However, DXA analysis revealed a significant reduction in bone area values (8.3% at 7 months, *P* < 0.01; Fig. 6D), in bone mineral content (9.8% at 4 months, *P* < 0.05; 12.9% at 7 months, *P* < 0.001; Fig. 6E) and in bone mineral density values (5.8% at 4 months, *P* < 0.05; 6% at 7 months, *P* < 0.05; Fig. 6F) in HNF4α^ΔIEC^ mice when compared to controls. Altogether, these data support that HNF4α^ΔIEC^ mice retain normal physiology under standard conditions except for altered bone density, a well-described osteotropic effects of GIP deficiency *in vivo*.

## Discussion

Incretins have long been recognized to play a central role in controlling the nutrition metabolism axis. Numerous reports have linked incretins to a number of metabolic features including fat metabolism and obesity^39,40^, bone metabolism^41^ and diabetes^42^. To date, only a small number of transcription factors have been reported to regulate GIP synthesis. GATA-4 regulate GIP expression in cell lines^37^ and Forkhead box protein O1 (FoxO1) as well as LEF1/β-catenin transcriptional complex mediate glucose and insulin-positive GIP regulation^43^. The regulatory factor X6 (Rfx6) acts as a positive regulator of GIP^44^ and coincidently, *RFX6* haploinsufficiency was found to be associated with a reduction of GIP in MODY^45^. The present findings further identify an additional disease-relevant regulatory connection between the transcription factor HNF4α, for which mutations cause MODY1, and the intestinal specific regulation of GIP. Since *MODY1* (*HNF4A*) mutations are somatic, concomitant alterations occurring in other tissues expressing HNF4α such as the intestinal epithelium are thus likely to be functionally involved during the onset of this disease. In support of this, patients with MODY1 showed a correlation between the total of insulin secretion during a test meal and GIP secretion^46^. In addition, a recent case report described an *HNF4A* mutation associated with an impaired incretin response during the progression of the MODY phenotype^47^. Our results open up for a more thorough investigation of this possible regulatory link in the context of MODY1 and other metabolic diseases.

Our results demonstrate that the loss of intestinal epithelial HNF4α impacts glucose-stimulated GIP production. Although a reduction of colonic GLP-1 producing cells was observed exclusively in the colon of HNF4α^ΔIEC^ mice, this observation did not significantly impact GLP-1 circulating levels under these conditions. It is possible that other GLP-1 producing sources may contribute to maintain homeostatic circulating GLP-1 levels in HNF4α^ΔIEC^ mice. HNF4α possible implication in the regulation of GLP-1 appears complex and will require further investigations. However, no significant difference in glucose tolerance was observed in HNF4α^ΔIEC^ mice, an observation reminiscent of the acute loss of K (GIP+) enteroendocrine cells in transgenic mice^48^. In addition, single deletion of the GIP receptor was found to have only a modest impact on glucose homeostasis in mice^49,50^. Glucose homeostasis is complex and involves several hormones and factors produced by various tissues. The impact of our findings on glucose homeostasis will have to be measured in multi-tissues knockout models. However, our model based on the loss of HNF4α in the intestinal epithelium recapitulates the findings made in various mouse models impaired for GIP signaling where bone metabolism was the most consistent physiological impairments observed in these mice^51–53^.

In conclusion, the present study allowed identifying a novel positive regulatory link between HNF4α (MODY1) and GIP incretin in the intestine and paves the way for further studies as to whether this molecular relationship may be therapeutically exploited in metabolism disorders including diabetes.

## Supplementary information

supplementary figures

## References

[1] Duncan SA (1994). Expression of transcription factor HNF-4 in the extraembryonic endoderm, gut, and nephrogenic tissue of the developing mouse embryo: HNF-4 is a marker for primary endoderm in the implanting blastocyst. Proc Natl Acad Sci USA.

[2] Sladek FM, Zhong WM, Lai E, Darnell JE (1990). Liver-enriched transcription factor HNF-4 is a novel member of the steroid hormone receptor superfamily. Genes Dev.

[3] Taraviras S, Monaghan AP, Schutz G, Kelsey G (1994). Characterization of the mouse HNF-4 gene and its expression during mouse embryogenesis. Mech Dev.

[4] Chen WS (1994). Disruption of the HNF-4 gene, expressed in visceral endoderm, leads to cell death in embryonic ectoderm and impaired gastrulation of mouse embryos. Genes Dev.

[5] Parviz F (2003). Hepatocyte nuclear factor 4alpha controls the development of a hepatic epithelium and liver morphogenesis. Nat Genet.

[6] Hayhurst GP, Lee YH, Lambert G, Ward JM, Gonzalez FJ (2001). Hepatocyte nuclear factor 4alpha (nuclear receptor 2A1) is essential for maintenance of hepatic gene expression and lipid homeostasis. Mol Cell Biol.

[7] Garrison WD (2006). Hepatocyte nuclear factor 4alpha is essential for embryonic development of the mouse colon. Gastroenterology.

[8] Babeu JP, Darsigny M, Lussier CR, Boudreau F (2009). Hepatocyte nuclear factor 4alpha contributes to an intestinal epithelial phenotype *in vitro* and plays a partial role in mouse intestinal epithelium differentiation. Am J Physiol Gastrointest Liver Physiol.

[9] Yamagata K (1996). Mutations in the hepatocyte nuclear factor-4alpha gene in maturity-onset diabetes of the young (MODY1). Nature.

[10] Fajans SS, Bell GI (2011). MODY: history, genetics, pathophysiology, and clinical decision making. Diabetes Care.

[11] Gupta RK (2005). The MODY1 gene HNF-4alpha regulates selected genes involved in insulin secretion. J Clin Invest.

[12] Miura A (2006). Hepatocyte nuclear factor-4alpha is essential for glucose-stimulated insulin secretion by pancreatic beta-cells. J Biol Chem.

[13] Stoffel M, Duncan SA (1997). The maturity-onset diabetes of the young (MODY1) transcription factor HNF4alpha regulates expression of genes required for glucose transport and metabolism. Proc Natl Acad Sci USA.

[14] Dhe-Paganon S, Duda K, Iwamoto M, Chi YI, Shoelson SE (2002). Crystal structure of the HNF4 alpha ligand binding domain in complex with endogenous fatty acid ligand. J Biol Chem.

[15] Hertz R, Magenheim J, Berman I, Bar-Tana J (1998). Fatty acyl-CoA thioesters are ligands of hepatic nuclear factor-4alpha. Nature.

[16] Yuan X (2009). Identification of an endogenous ligand bound to a native orphan nuclear receptor. PLoS One.

[17] Shih DQ (2000). Genotype/phenotype relationships in HNF-4alpha/MODY1: haploinsufficiency is associated with reduced apolipoprotein (AII), apolipoprotein (CIII), lipoprotein(a), and triglyceride levels. Diabetes.

[18] Yin L, Ma H, Ge X, Edwards PA, Zhang Y (2011). Hepatic hepatocyte nuclear factor 4alpha is essential for maintaining triglyceride and cholesterol homeostasis. Arterioscler Thromb Vasc Biol.

[19] Marcil V (2010). Modification in oxidative stress, inflammation, and lipoprotein assembly in response to hepatocyte nuclear factor 4alpha knockdown in intestinal epithelial cells. J Biol Chem.

[20] Frochot V (2012). The transcription factor HNF-4alpha: a key factor of the intestinal uptake of fatty acids in mouse. Am J Physiol Gastrointest Liver Physiol.

[21] Yabe D, Seino Y (2013). Incretin actions beyond the pancreas: lessons from knockout mice. Curr Opin Pharmacol.

[22] Madison BB (2002). Cis elements of the villin gene control expression in restricted domains of the vertical (crypt) and horizontal (duodenum, cecum) axes of the intestine. J Biol Chem.

[23] Darsigny M (2009). Loss of hepatocyte-nuclear-factor-4alpha affects colonic ion transport and causes chronic inflammation resembling inflammatory bowel disease in mice. PLoS One.

[24] Darsigny M (2010). Hepatocyte nuclear factor-4alpha promotes gut neoplasia in mice and protects against the production of reactive oxygen species. Cancer Res.

[25] Brial F, Lussier CR, Belleville K, Sarret P, Boudreau F (2015). Ghrelin Inhibition Restores Glucose Homeostasis in Hepatocyte Nuclear Factor-1alpha (MODY3)-Deficient Mice. Diabetes.

[26] Lacraz G (2016). Deficiency of Interleukin-15 Confers Resistance to Obesity by Diminishing Inflammation and Enhancing the Thermogenic Function of Adipose Tissues. PLoS One.

[27] Ferrannini E (1988). The theoretical bases of indirect calorimetry: a review. Metabolism.

[28] Peronnet F, Massicotte D (1991). Table of nonprotein respiratory quotient: an update. Can J Sport Sci.

[29] Boudreau F (2007). Loss of cathepsin L activity promotes claudin-1 overexpression and intestinal neoplasia. FASEB J.

[30] Langlois MJ (2009). Epithelial phosphatase and tensin homolog regulates intestinal architecture and secretory cell commitment and acts as a modifier gene in neoplasia. FASEB J.

[31] Lepage D (2015). Identification of GATA-4 as a novel transcriptional regulatory component of regenerating islet-derived family members. Biochim Biophys Acta.

[32] Frechette I, Darsigny M, Brochu-Gaudreau K, Jones C, Boudreau F (2010). The Promyelocytic Leukemia Zinc Finger (PLZF) gene is a novel transcriptional target of the CCAAT-Displacement-protein (CUX1) repressor. FEBS J.

[33] Cartharius K (2005). MatInspector and beyond: promoter analysis based on transcription factor binding sites. Bioinformatics.

[34] Boudreau F (2002). Hepatocyte nuclear factor-1 alpha, GATA-4, and caudal related homeodomain protein Cdx2 interact functionally to modulate intestinal gene transcription. Implication for the developmental regulation of the sucrase-isomaltase gene. J Biol Chem.

[35] Suzuki K (2018). Distribution and hormonal characterization of primary murine L cells throughout the gastrointestinal tract. J Diabetes Investig.

[36] Boylan MO, Jepeal LI, Jarboe LA, Wolfe MM (1997). Cell-specific expression of the glucose-dependent insulinotropic polypeptide gene in a mouse neuroendocrine tumor cell line. J Biol Chem.

[37] Jepeal LI, Boylan MO, Michael Wolfe M (2008). GATA-4 upregulates glucose-dependent insulinotropic polypeptide expression in cells of pancreatic and intestinal lineage. Mol Cell Endocrinol.

[38] Sumi K (2007). Cooperative interaction between hepatocyte nuclear factor 4 alpha and GATA transcription factors regulates ATP-binding cassette sterol transporters ABCG5 and ABCG8. Mol Cell Biol.

[39] Miyawaki K (2002). Inhibition of gastric inhibitory polypeptide signaling prevents obesity. Nat Med.

[40] Ding X, Saxena NK, Lin S, Gupta NA, Anania FA (2006). Exendin-4, a glucagon-like protein-1 (GLP-1) receptor agonist, reverses hepatic steatosis in ob/ob mice. Hepatology.

[41] Ramsey W, Isales CM (2017). Intestinal Incretins and the Regulation of Bone Physiology. Adv Exp Med Biol.

[42] Gamble JM (2015). Incretin-based medications for type 2 diabetes: an overview of reviews. Diabetes Obes Metab.

[43] Garcia-Martinez JM, Chocarro-Calvo A, De la Vieja A, Garcia-Jimenez C (2014). Insulin drives glucose-dependent insulinotropic peptide expression via glucose-dependent regulation of FoxO1 and LEF1/beta-catenin. Biochim Biophys Acta.

[44] Suzuki K (2013). Transcriptional regulatory factor X6 (Rfx6) increases gastric inhibitory polypeptide (GIP) expression in enteroendocrine K-cells and is involved in GIP hypersecretion in high fat diet-induced obesity. J Biol Chem.

[45] Patel KA (2017). Heterozygous RFX6 protein truncating variants are associated with MODY with reduced penetrance. Nat Commun.

[46] Ekholm E, Shaat N, Holst JJ (2012). Characterization of beta cell and incretin function in patients with MODY1 (HNF4A MODY) and MODY3 (HNF1A MODY) in a Swedish patient collection. Acta Diabetol.

[47] Arya VB (2014). HNF4A mutation: switch from hyperinsulinaemic hypoglycaemia to maturity-onset diabetes of the young, and incretin response. Diabet Med.

[48] Pedersen J (2013). Glucose metabolism is altered after loss of L cells and alpha-cells but not influenced by loss of K cells. Am J Physiol Endocrinol Metab.

[49] Miyawaki K (1999). Glucose intolerance caused by a defect in the entero-insular axis: a study in gastric inhibitory polypeptide receptor knockout mice. Proc Natl Acad Sci USA.

[50] Pamir N (2003). Glucose-dependent insulinotropic polypeptide receptor null mice exhibit compensatory changes in the enteroinsular axis. Am J Physiol Endocrinol Metab.

[51] Xie D (2005). Glucose-dependent insulinotropic polypeptide receptor knockout mice have altered bone turnover. Bone.

[52] Mieczkowska A, Irwin N, Flatt PR, Chappard D, Mabilleau G (2013). Glucose-dependent insulinotropic polypeptide (GIP) receptor deletion leads to reduced bone strength and quality. Bone.

[53] Mansur SA (2015). Stable Incretin Mimetics Counter Rapid Deterioration of Bone Quality in Type 1 Diabetes Mellitus. J Cell Physiol.

