# Supplementary material for: HNF4α is a novel regulator of intestinal glucose-dependent insulinotropic polypeptide
Source: Sci Rep. 2019 Mar 12;9:4200. doi: 10.1038/s41598-019-41061-z (PMC6414548; doi:10.1038/s41598-019-41061-z)
Supplement: Supplementary file 1 — supplementary figures [file 41598_2019_41061_MOESM1_ESM.pdf]

HNF4α is a novel regulator of intestinal glucose-dependent insulinotropic polypeptide

Romain Girard[1], Mathieu Darsigny[1], Christine Jones[1], Faïza Maloum-Rami[1], Yves Gélinas[2], André C. Carpentier[3], Mathieu Laplante[2], Nathalie Perreault[1], François Boudreau[1*]

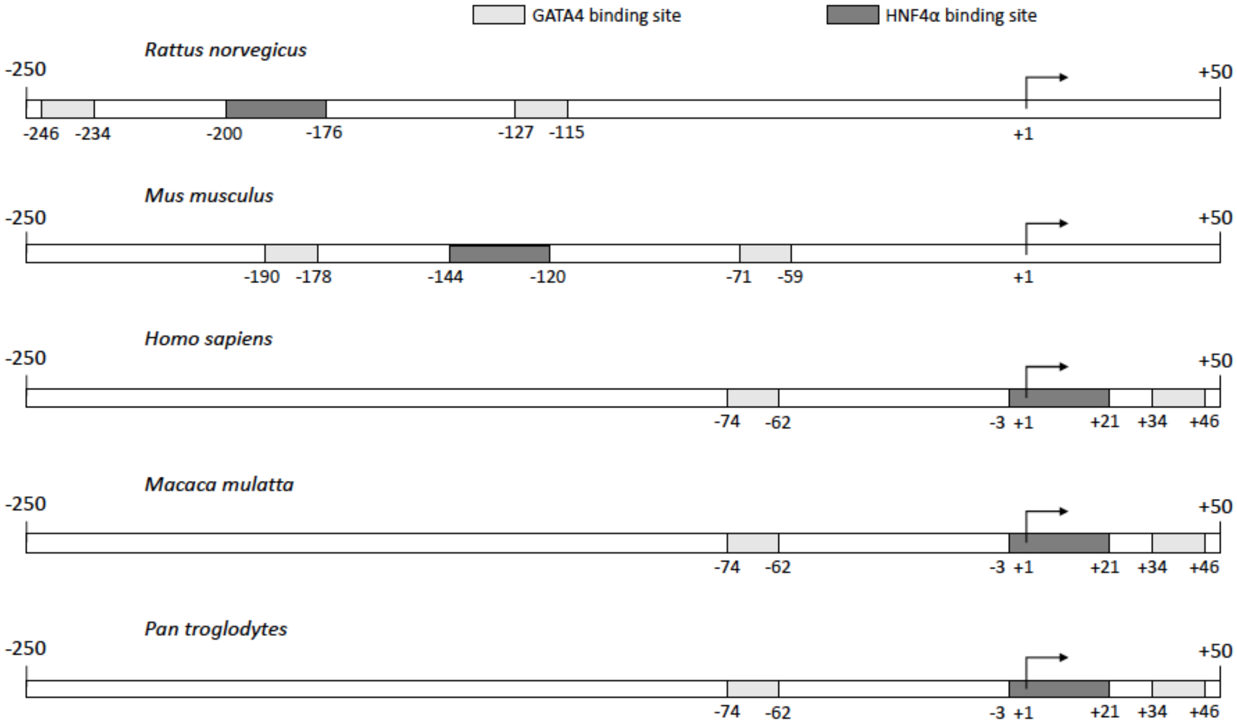

Supplementary Figure 1. Promoter analysis of GIP among several species with the use of the MatInspector software tool, http://www.genomatix.de.

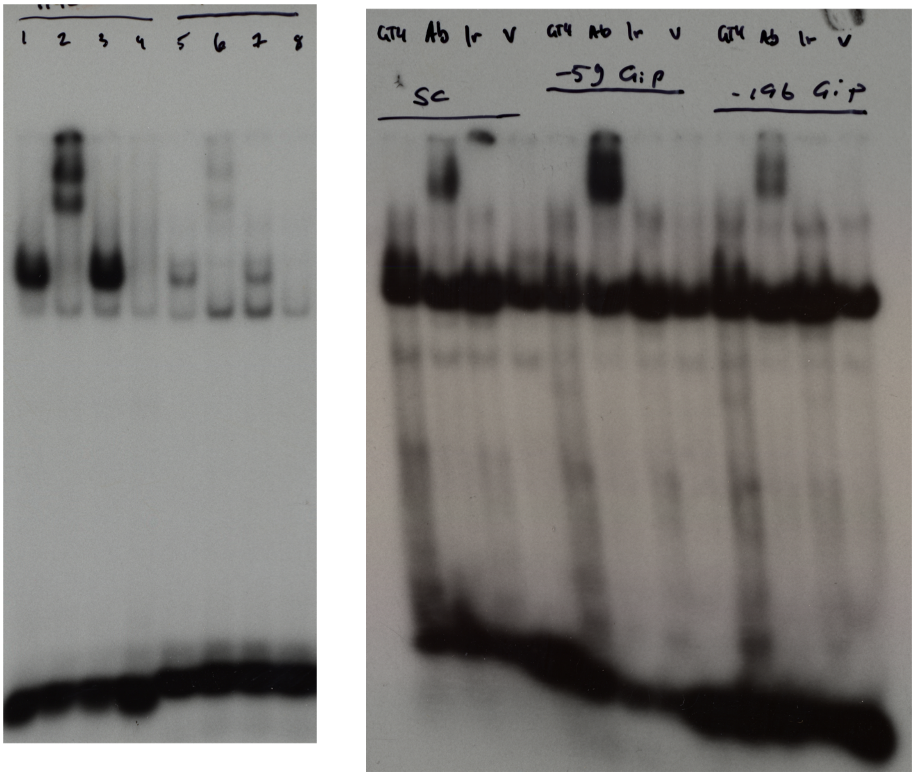

Supplementary Figure 2. Full length gels of EMSA for HNF4alpha interaction with DNA (left panel) and for GATA4 interaction with DNA (right panel).
